# Toxic Organic Contaminants in Airborne Particles: Levels, Potential Sources and Risk Assessment †

**DOI:** 10.3390/ijerph18084352

**Published:** 2021-04-20

**Authors:** Donatella Pomata, Patrizia Di Filippo, Carmela Riccardi, Federica Castellani, Giulia Simonetti, Elisa Sonego, Francesca Buiarelli

**Affiliations:** 1 Italian Workers’ Compensation Authority via Roberto Ferruzzi 38, 00143 Rome, Italy; d.pomata@inail.it (D.P.); p.difilippo@inail.it (P.D.F.); ca.riccardi@inail.it (C.R.); 2 Department of Chemistry, Sapienza University of Rome, 00185 Rome, Italy; federica.castellani@uniroma1.it (F.C.); elisa.sonego@uniroma1.it (E.S.); francesca.buiarelli@uniroma1.it (F.B.)

**Keywords:** organic pollutant measurements, particle-size distribution, outdoor environments, principal component analysis

## Abstract

In the last years, many studies have focused on risk assessment of exposure of workers to airborne particulate matter (PM). Several studies indicate a strong correlation between PM and adverse health outcomes, as a function of particle size. In the last years, the study of atmospheric particulate matter has focused more on particles less than 10 μm or 2.5 μm in diameter; however, recent studies identify in particles less than 0.1 μm the main responsibility for negative cardiovascular effects. The present paper deals with the determination of 66 organic compounds belonging to six different classes of persistent organic pollutants (POPs) in the ultrafine, fine and coarse fractions of PM (PM < 0.1 µm; 0.1 < PM < 2.5 µm and 2.5 < PM < 10 µm) collected in three outdoor workplaces and in an urban outdoor area. Data obtained were analyzed with principal component analysis (PCA), in order to underline possible correlation between sites and classes of pollutants and characteristic emission sources. Emission source studies are, in fact, a valuable tool for both identifying the type of emission source and estimating the strength of each contamination source, as useful indicator of environment healthiness. Moreover, both carcinogenic and non-carcinogenic risks were determined in order to estimate human health risk associated to study sites. Risk analysis was carried out evaluating the contribution of pollutant distribution in PM size fractions for all the sites. The results highlighted significant differences between the sites and specific sources of pollutants related to work activities were identified. In all the sites and for all the size fractions of PM both carcinogenic and non-carcinogenic risk values were below acceptable and safe levels of risks recommended by the regulatory agencies.

## 1. Introduction

Air pollution in urban and industrial areas causes serious damage to human health, including hospitalization for respiratory and cardiovascular diseases, and aggravation of asthma attacks, adverse lung functions and mortality [1,2,3,4]. In recent years, many epidemiological and toxicological studies have examined the inhalation of airborne particulate matter and particle dose and deposition in human airways, depending on the size [5]. The smaller the size and the higher the content in toxicants (i.e., heavy metals, polycyclic aromatic hydrocarbons (PAHs), quinones and endotoxins) the more hazardous the PM is.

PM is usually classified in PM10 and PM2.5, including particles with aerodynamic diameter smaller than 10 and 2.5 µm, respectively. Besides these two fractions, recent studies have focused on particles smaller than 1 µm (PM 1—ultrafine particles), since they can more easily penetrate the alveolar region. Epidemiological studies indeed confirm a higher deposition of ultrafine particles in the alveolar region and a higher capability to enter the bloodstream, affecting inner organs and penetrate the brain [6,7]. In addition, the ultrafine particles have a large effective surface area, which allows them to act as carriers of harmful chemicals [8] making the magnitude of their contribution on the human health currently unclear.

In this optic, the study of chemical composition of PM, including ultrafine dimensional fractions and coarse and fine particles, could not only enhance the identification of different emission sources, characteristic of specific environments, but also provide important insight into their contribution to adverse human health effects. In particular, outdoor environments studies are more complex since they are characterized by numerous emission sources of PM, both anthropogenic and of natural origin. The most common anthropogenic sources are combustion processes used for electricity generation, transport, industry and households, industrial processes and solvent use, agricultural crops and livestock, waste incinerator and wastewater treatment plant. Polluting vehicle emissions constitute the major contribution to the deterioration of urban air quality. In addition, black carbon (BC), emitted from the combustion of fossil fuels due to the low efficiency of residential heating systems, contributes largely to poor air quality and to global warming [9,10].

Instead, in workplace areas, identification of pollution source is more challenging because of the synergic interaction between outdoor emission and specific sources, due to particles emitted in processes typical of each work activity. The British Safety Council (BSC), in the Time to Breathe campaign [11], highlights a growing concern about harm to health from exposure to air pollution for outdoor workers. In this regard, risk assessment, carried out using the calculation of carcinogenic and non-carcinogenic risk as suggested by USEPA [12], is a fast tool for evaluating the effects of exposure to hazards on human health. Several studies are focused on assessing the risk of exposure to heavy metals for workers involved in activities where the emission of these compounds is predominant [13,14]. On the contrary, a challenging task is represented by the identification of organic compound sources in outdoor workplaces where multiple emission sources can coexist since related to the specific work activities and the surrounding environment. In this optic, the chemical characterization of PM and the evaluation of the contribution of the different aerosol sources can be the basis for assessing the public health risk due to exposure to particle-bound pollutants and planning efficient strategies of air quality management [15]. Therefore, the aim of the present study was to determine concentrations of 66 organic pollutants, collected in three workplaces and in an urban outdoor area in order to identify characteristic or common emission sources, responsible for the presence of toxic contaminants in the monitoring areas. Data obtained were analyzed with principal component analysis (PCA) to obtain source apportionment results as suggested by many authors [15,16,17,18]. In addition, both carcinogenic and non-carcinogenic risk were determined in order to estimate human health risk associated to study sites. Risk analysis was carried out evaluating the contribution of pollutant distribution in size-fractionated particles and the related inhalation risk to which workers are daily exposed.

The results highlight that an accurate study of source identification has to be performed to characterize specific sources of pollutants related to work activities. Furthermore, the risk assessment, carried out to determine pollutant concentrations in PM size fractions, further contributes to site characterization and to the evaluation of environment healthiness. Therefore, due to the simultaneous study of source identification and risk assessment, it is possible to plan suitable actions for pollution prevention and risk management.

## 2. Materials and Methods

### 2.1. Sampling

**Figure 1:**
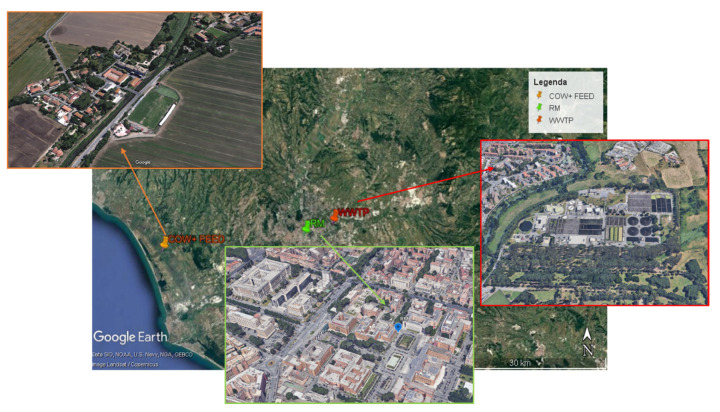
Location map of monitoring sites: one outdoor urban area (RM) and three outdoor industrial workplaces (WWTP, COW and FEED).

Airborne particles of different sizes were collected in four monitoring areas located in Central Italy (Figure 1). Table 1 shows the likely sources of pollutants in the study sites. The urban site (RM) is characterized mainly by anthropogenic activities. The wastewater treatment plant (WWTP) is an industrial site located in a suburban area therefore, it is influenced by both urban and industrial sources. The shed (COW) and the feed (FEED) areas are two industrial sites located in a rural area influenced by work activities of livestock facility.

Particle collection was performed from 21 March to 30 April 2016 in RM and WWTP areas and from 21 March to 30 April 2018 in an intensive livestock farming activity located about 35 km from the center of Rome, where open barns house dairy and slaughter cows and beef calves. Airborne PM was sampled in two different sites within the farm, nearby a shed used as stables (named COW) and close to a shed for the grinding, mixing and storing of feeds (named FEED). Samplings were performed continuously during working hours. In all the monitoring areas the samplings were carried out in the spring period in order to obtain comparable results. Regardless of the sampling year, in all the sites the meteorological conditions were characterized by mild temperatures (13–18 °C) and low rainfall typical of Italian spring season.

DLPI samplers (Dekati Low Pressure Impactor, Dekati, Kangasala, Finland), consisting of thirteen stages (from 10,000 to 30 nm of aerodynamic diameter, Dp), operating at a flow rate of 10 L/min, were used to trap particles on polycarbonate (PCTE) membrane filters, 0.45 μm pore size and 25 mm diameter (Merck Millipore, Merck S.p.a., Vimodrone, Milan, Italy). Before and after the samplings, preconditioned filters (50% R.H. and 20 °C) were weighed in triplicate to within ± 0.001 mg, on a microbalance MC5 Sartorius, (Sartorius Lab Holding GmbH, Goettingen, Germany). After, the thirteen filters from each sampler were reassembled into three sets: ultrafine fraction that corresponded to particles with 0.016 < Da < 0.094 μm; fine fraction that corresponded to particles with 0.15 < Da < 2.46 and finally coarse fraction that corresponded to particles with 3.63 < Da < 10.

### 2.2. Analytical Methods

The analytical procedure was described in detail in previous studies [19,20,21]. Briefly, filters were extracted by an accelerated solvent extractor ASE 200 (Dionex, Sunnyvale, CA, USA) with DCM/n-Hexane (1:1) (two cycles) followed by ethyl acetate (two cycles) at 100 °C and 1500 psi. Extract clean-up was carried out simultaneously to the extraction, filling ASE cells with florisil as sorbent.

The extracts were evaporated and redissolved with 50 μL of toluene until analytical determinations.

The analysis of Polychlorinated Biphenyls (PCBs), Polybrominated diphenyl ethers (PBDEs), Novel Brominated Flame Retardants (NBFRs), Polycyclic Aromatic Hydrocarbons (PAHs) and PAH derivatives (oxy-PAHs and nitro-PAHs), reported in the Supplementary Materials (Table S1), was performed by gas chromatography–mass spectrometry (GC–MS). An HP 7890-B gas chromatograph fitted with an HP 7693 autosampler and coupled with an HP 5977B single quadrupole mass-selective detector (Agilent Technologies, Palo Alto, CA, USA) was used for GC–MS analysis both in electronic ionization (EI) and in negative chemical ionization (NCI). The temperature program in GC-EI–MS (operating at 70 eV) and in GC-NCI–MS (using methane as a reagent gas at 40 mL/min) was: 100 °C initial temperature, ramped at 25 °C/min to 310 °C and held for 8 min. The quadrupole was set at 150 °C, the ion source temperatures were set at 230 °C and the transfer line at 300 °C. The injector temperature was set at 280 °C and the samples (1 µL) were injected in the splitless mode. The helium carrier gas was set at a constant flow rate of 1.0 mL/min. The instrument was tuned using the software autotune procedure (Agilent MSD Chem Station D.01.00 software) (Agilent Technologies Italia S.p.A., Cernusco sul Naviglio MI, Italy) and selective ion monitoring (SIM) was used in both MS configurations.

The quantitative analysis of target compounds was performed based on matrix matched calibration curves built by using parallel samplings. Each of the three extracts from ultrafine, fine and coarse particles were split in five aliquots and added with standard solutions of all analytes at growing concentrations. The results were subtracted of the environmental concentration and the three resulting curves, for each PM fraction, were used as matrix matched calibration curves.

### 2.3. Human Health Risk Assessment

In agreement with Mustafa et al. [22] and as described in the USEPA Risk Assessment Guidance for Superfund [12], The exposure concentration for an individual compound ECi (mg/m^3^) was calculated using the following equations

(1) $$\mathrm{ECi}=\frac{\mathrm{Ci}*\mathrm{DET}*\mathrm{EF}*\mathrm{ED}}{\mathrm{AT}\;*\;365\;*\;24\;*\;1000}$$

where Ci is the emission concentration for each compound (μg/m^3^), DET is the daily exposure time (8 h/d), EF is the exposure frequency (350 d × y), ED is the exposure duration (30 y) and AT is the average time (25 y for non-carcinogenic risks and 70 y for carcinogenic risks).

The non-carcinogenic risk (HR) was calculated using Equation (2), in which RFC is the inhalation reference concentration (mg/m^3^). The carcinogenic risk (CR) was calculated using Equation (3), in which IUR is the inhalation unit risk value (μg/m^3^)^−1^. The IUR values was taken from U.S.EPA [12]

(2) $$\mathrm{HR}=\frac{{\mathrm{EC}}_{i}}{\mathrm{RFC}}$$

(3) $$\mathrm{CR}={\mathrm{EC}}_{i}\;*\;\mathrm{IUR}\;*\;1000$$

Non-carcinogenic (HR) and carcinogenic (CR) risks were evaluated for the target compounds using parameter values reported in Tables S2–S7

CR was estimated for PAHs, Oxy- and Nitro-PAHs, and PCB in both PM10 and ultrafine, fine and coarse fractions. However, since for some analytes, parameter values are lacking, the human health risk was determined using the data of compound with similar chemical–physical characteristics or belonging to same class of contaminants and with comparable toxicity. Total carcinogenic risk was calculated by summing the individual risks obtained for every compound class in the three PM fractions. Total CR was compared to values recommended by USEPA [12] that, for public health protection, suggests CR < 1 × 10^−6^ as acceptable risk level and < 1 × 10^−4^ as a tolerable risk level [23,24].

HR was calculated for PBDE and some NBFRs that have similar potentials for bioaccumulation, persistence and atmospheric transport as PBDEs [25]. Total non-carcinogenic risk was obtained by summing the HR values of every compound class and comparing them to those suggested from USEPA [12]. If HR < 1 no appreciable risk of non-carcinogenic effects may occur, while HR > 1 indicates chance of non-carcinogenic effects [23].

## 3. Results and Discussion

### 3.1. PM Mass Concentration

**Figure 2:**
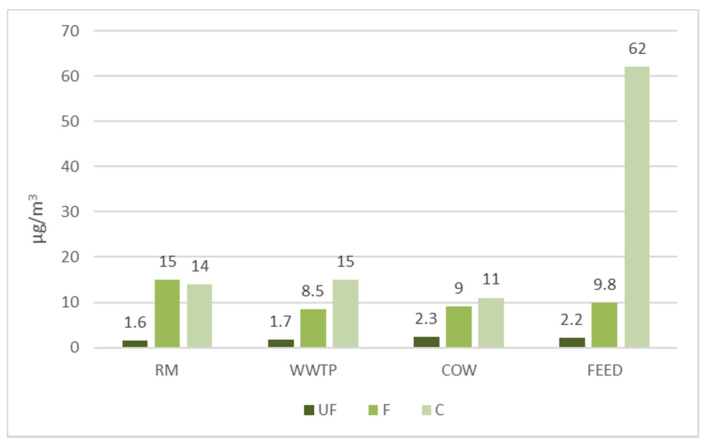
Mass concentration of ultrafine (UF), fine (F) and coarse (C) airborne particles in the urban site (RM) and in the three workplaces (WWTP: Wastewater treatment plant. COW: Shed site. FEED: Feed site).

Figure 2 shows the mass concentration values (µg/m^3^) of ultrafine (UF), fine (F) and coarse (C) particles sampled in each site calculated as average of the whole monitoring period. UF concentrations are similar in the four monitored environments.

A double concentration of F is found in the urban area. This fraction is typically higher in urban areas than in suburban and rural areas due to prevalence of sources such as traffic and residential housing emission [26]. In addition, the percentage of fine particle mass on the total PM is about 50% in agreement with other authors [21] that detected comparable values in outdoor atmospheres.

The higher concentration of C is found in FEED where the constant grinding of the feed causes a high rising of dust with a larger aerodynamic diameter. Furthermore, it is known that the agricultural and livestock industry generates several milligrams per hour of PM with much larger mass median diameters than urban dusts [27]. 

### 3.2. Organic Compound Concentrations

**Figure 3:**
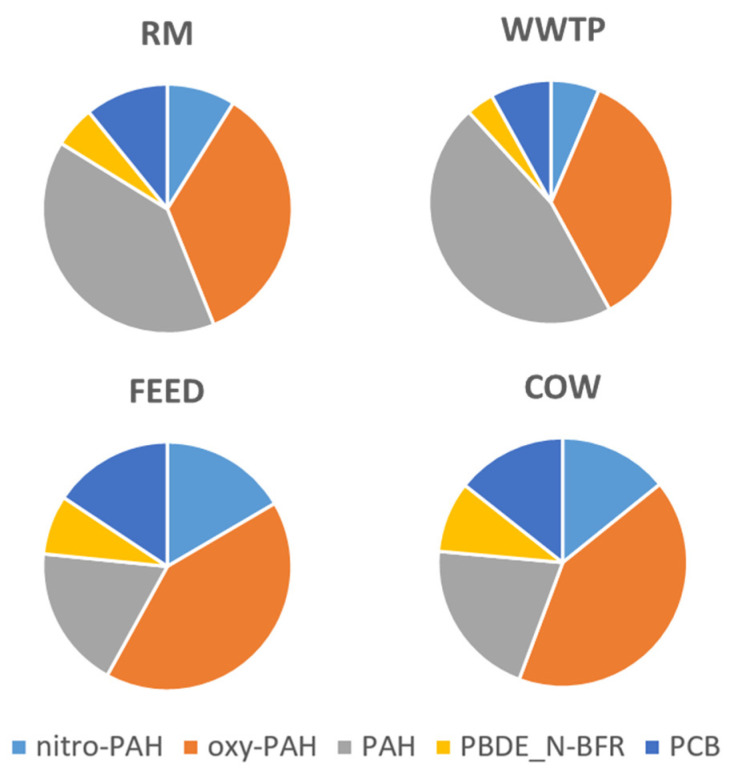
Pie chart reporting organic contaminant group concentrations in ng per mg of PM10, in the four sampling sites.

Figure 3 compares the four sampling sites for the distribution of the classes of compounds in PM10 assuming a common source for PBDE and NBFR.

The results highlight the higher presence of PAH in the two sites RM and WWTP. Oxy- and nitro- PAHs, consisting of pollutants as they are emitted but also coming from atmospheric transformations, are higher in the farm, probably due to pollutants coming from the city, having the time to undergo chemical reactions in the atmosphere during their path [28]. PCB, PBDE and NBFR are also higher in the farm samples, probably due to additional sources in this site, on respect the city and the wastewater treatment plant. This finding can be due to initial feed contamination since both feed and seeds are often contaminated by flame retardant [29]. In this site, the work activities, such as the grinding and handling of the feed, were routinely carried out.

**Figure 4:**
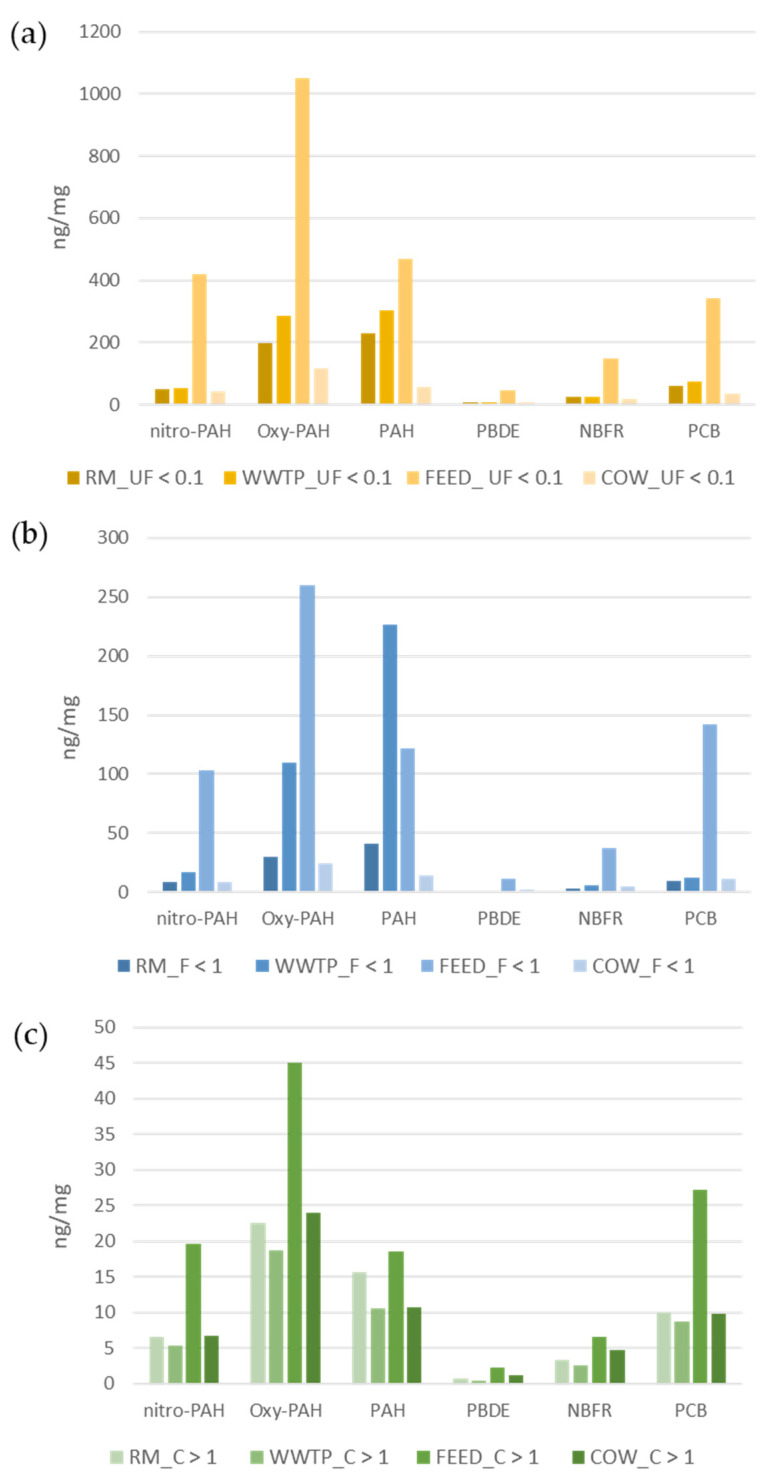
Distribution (ng per mg of PM) of all groups of organic substances in ultrafine (**a**), fine (**b**) and coarse (**c**) fractions of PM.

Figure 4 shows the distribution of all groups of organic substances in the three fractions of PM. All classes of compounds, except for PAHs in WWTP site, have a higher concentration per unit of PM at the FEED site in all the three particulate fractions; as regards UF, F and C fractions, the distribution of organic substances is proportional, but the finer is the fraction, the more concentrated are the organic substances [30].

As expected, PAH and derivatives contribute more significantly to the organic composition of particles, than other classes of compounds, due to a higher number of emission sources. In particular, photochemical production is the source of the majority of nitro- and oxy- PAH, as evidenced by the higher concentrations of those compounds produced in the atmosphere as secondary pollutants: 9- 2- and 3- nitro- phenanthrenes and 9-fluorenone in all the PM fractions, and in all the sites.

Among, NBFR and PCB, TBECH and TBPH and PCB28, respectively, are the most abundant in all the fractions of PM and in all the sites.

**Figure 5:**
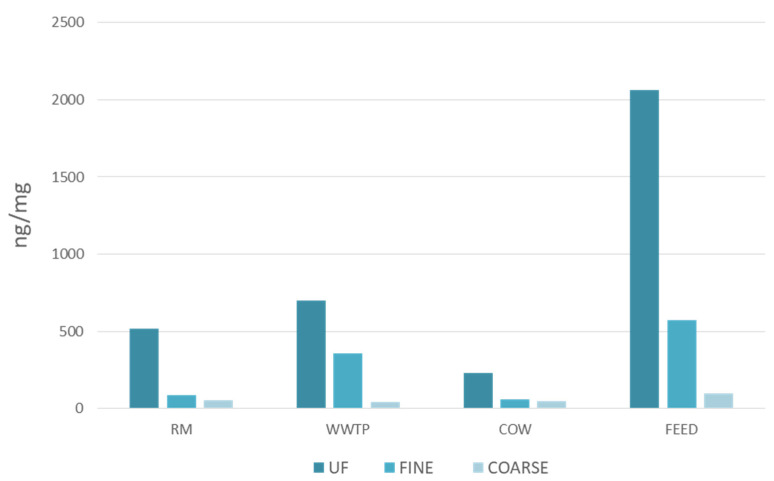
Distribution of the toxics analyzed in the three fractions of PM in each sampling site.

Figure 5 shows the distribution of the toxics analyzed in the three fractions of PM in each sampling site. Organic compound content of particles was determined as the sum of ng toxic substances in relation to amount (mg) of PM in each fraction. A higher percentage of analytes target was observed in the smaller fractions. PM concentrations are the higher, the greater the size is, but ultrafine particles are richer in organic compounds. Therefore, the concentration (ng/mg) of toxic substances is higher in ultrafine fraction (lower dust amount) than the one in the coarse fraction, despite this fraction is characterized by the highest mass concentration. Thus, not only ultrafine particles penetrate deeper in human respiratory system but they proportionally transmit a greater number of toxic substances.

### 3.3. Principal Component Analysis (PCA)

Multivariate statistical computations were performed using the statistical software R (R-project for statistical computing, Ver. 3.0, 32-bit, Vienna, Austria). A principal component analysis (PCA) was calculated on the matrix of data of Figure 4, including the chemical concentrations (ng per mg of PM) of 14 PAHs, 9 Ox-PAHs, 10 Nitro-PAHs, 15 PCB, 7 PBDE and 11 NBFR, in the three PM dimensional fractions (UF < 1, F > 1 and C < 10 µm), in the four different sampling sites. The data matrix was transformed by performing row and column autoscaling to overcome the scale variations between the examined variables [31]. Two principal component analyses were run on all compounds, and on the individual classes of contaminants (PAHs and derivatives, PCBs, PBDEs and NBFRs). 

In Supplementary Material, Table S1 lists compound and site codes used in PCA development.

#### 3.3.1. All Organic Compounds

**Figure 6:**
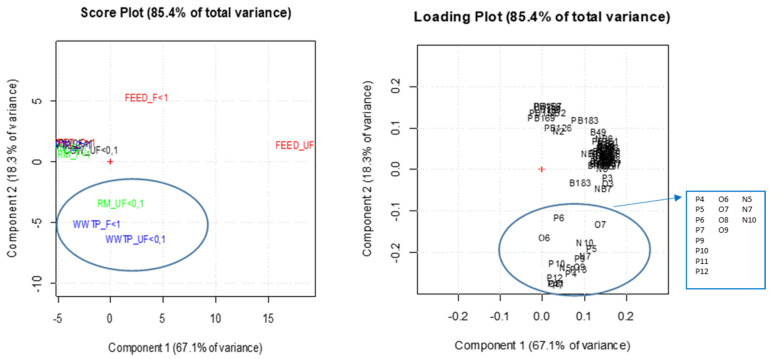
Loading plot and the score plot (left and right respectively) for all target compounds in all sampling sites and in the three PM dimensional fractions. Compound codes, listed in the side square, are identified in the Supplementary Materials (Table S1).

Figure 6 shows the loading plot and the score plot for all target compounds in all sampling sites and in the three PM dimensional fractions. The two main components PC1 and PC2 explained 85.4% of total variance. The score plot highlights a well-defined cluster, including WWTP F < 1 and UF < 0.1 and RM UF < 0.1, that shows a significant correlation with the group of compounds positioned at the bottom of the loading plot (blue circles). This area corresponds to data relating to some PAHs and derivatives characteristic of anthropogenic sources. Therefore, the pollution sources related to the surrounding urban area seem to strongly affect the WWTP site due to the urbanization impact of metropolises like Rome with high population density and anthropogenic activities [32].

Furthermore, FEED F < 1 and UF < 0.1 also seem to be influenced by specific contamination sources, even if different from those identified in WWTP and RM sites. However, it is not possible to find any correlation with specific compounds as, in the upper part of the loading plot, the compounds are unevenly distributed and no cluster is shown.

More information can be obtained by considering the PCA carried out on each of the three macroclasses of compounds (PAHs and derivatives, PCB, PBDE and NBFR).

#### 3.3.2. Individual Classes of Organic Compounds

**Figure 7:**
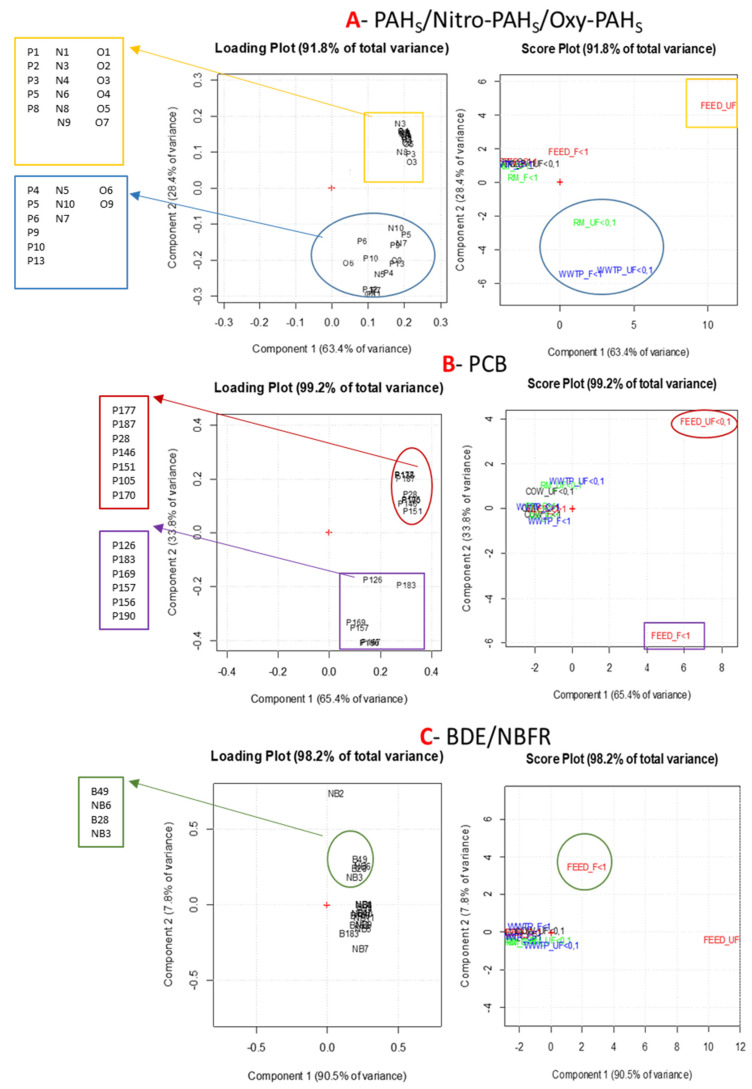
Loading and score plots of PCA carried out on the three classes of compounds: PAHs and derivatives (**A**), PCBs (**B**), PBDE and NBFR (**C**). Compound codes, listed in the side frames, are identified in the Supplementary Materials (Table S1).

Figure 7 shows loading and score plots for each single classes of compounds.

Plots relating to PAHs and derivatives (Figure 7A) highlight a significant correlation among WWTP F < 1, UF < 0.1, RM UF < 0.1 and the high molecular weight PAHs (HMWPAHs), some NO- and Oxy-PAHs (blue circles). As HMWPAHs have a pyrolytic origin, this result suggests combustion as the main source of contamination in WWTP and RM. The NO-PAHs that most affect the sites were 7NBaA and 9NA, both of primary origin and due to direct emissions from combustion processes, and 2NF of secondary origin by photochemical mechanisms. The good correlation with 9,10-Phenanthrene quinone, 1,9-Benz-10-anthron and BaA-7,12-dione, generated by both combustion processes and reactions in atmosphere, further supports the hypothesis of combustion as predominant source of pollution for WWTP and RM in the fine and ultrafine fractions of PM [20,21].

Lastly, the PCA, carried out with PAH and derivative data, also highlights that WWTP and RM sites are influenced by similar emission sources in agreement with their location. In the wastewater treatment plants, biological oxidation tanks can be considered the main aerosol source. However, since the site is located in a suburban area, PCA analysis highlighted that neighboring urban area is the main source of pollution and the aeration tanks contribute negligibly.

PCBs show a good correlation with FEED site for fine and ultrafine PM fraction (Figure 7B). FEED F < 1 correlates with 5 dioxin-like PCBs (126, 156, 157, 167 and 169) (purple square), while FEED UF < 0.1 is influenced by others PCBs including also PCB28 and 138 (red circles). These two compounds, with PCB52, 101, 153 and 180 are considered indicators of contamination in food and feed and their sum includes about half of the total amount of no dioxin-like PCBs detected in these matrices [33]. WWTP and RM sites do not seem to be influenced by PCB sources.

PBDE and NBFR classes show a significant correlation with FEED site for fine PM fraction (Figure 7C). A well-defined cluster is highlighted in the upper part of the loading plot (green circles) related to the FEED < 1. The compounds that characterize this group are BDE28 and 49, PBEBs and TBPs in agreement with Fernandes et al. [34] that have detected the presence of PBDEs and some NBFRs in animal feed at non-negligible concentrations. Additionally, for PCBs, WWTP and RM sites did not show any correlation with PBDE and NBFR.

## 4. Risk Assessment

### 4.1. PAHs 

**Figure 8:**
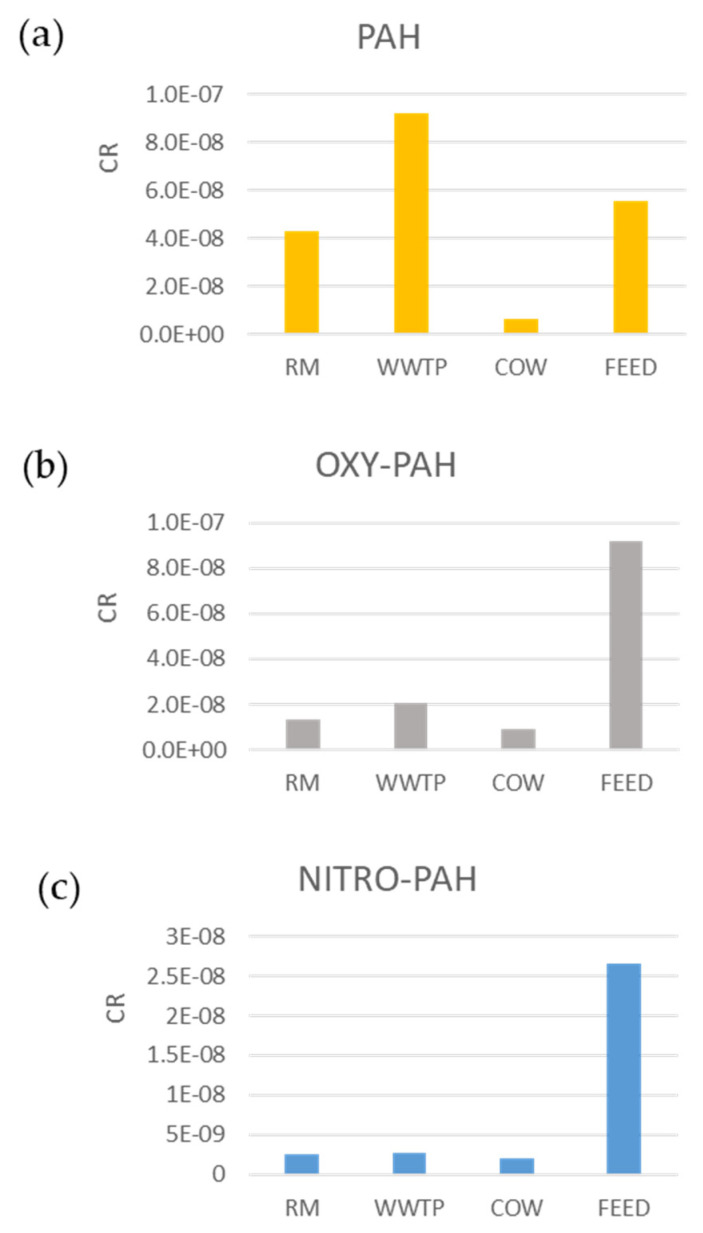
Carcinogenic risk from PAHs (**a**), OXY- (**b**) and NITRO-PAHs (**c**) in PM10 in the four sampling sites.

Figure 8 shows CR due to PM10 bound PAHs and derivatives in the study sites.

CR ranges from 6 × 10^−9^ to 9 × 10^−8^ for PAHs, from 9 × 10^−9^ to 9 × 10^−8^ for Oxy-PAHs and from 3 × 10^−9^ to 2.7 × 10^−8^ for Nitro-PAHs and all values are below the risk acceptable level (1 × 10^−6^). Nevertheless, significant differences can be observed among the sites. CR from PAHs is higher in WWTP, while Oxy- and Nitro-PAHs contribute to greater health risk in the FEED site. The COW site shows the lowest CR values for all classes of compounds. As demonstrated by PCA, in WWTP the highest CR values associated with PAHs may be due to the predominance of pyrolytic sources in agreement with site location. WWTP can be considered a suburban area characterized by both vehicular traffic and agricultural activities that require the use of industrial vehicles such as tractors, threshers, etc. In addition, some activities such as open burning of brushwood, straw and stubble are widely spread in the spring. CR in the FEED site is mainly affected by PAH derivatives from emissions of diesel and gasoline engines in accordance with work activities carried out in the area: seed grinding with motor mill and feed transport with industrial motor vehicles. Therefore, the higher values obtained may be due to emissions of diesel and gasoline engines of vehicles used to mill and move feed.

**Figure 9:**
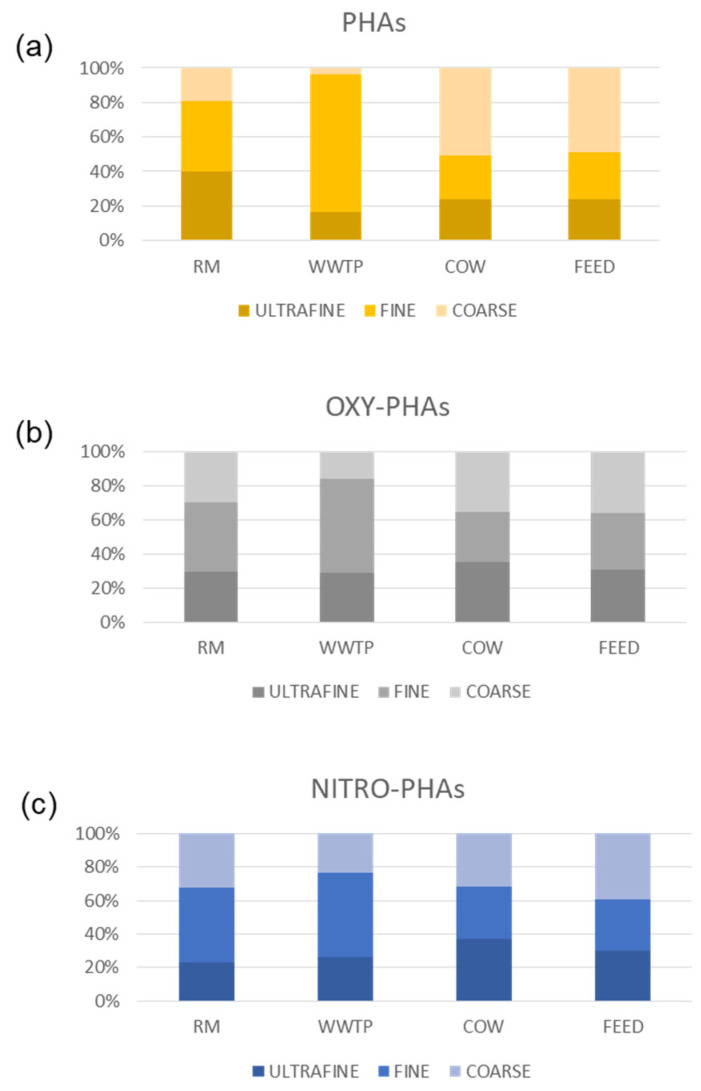
Percentage distribution of carcinogenic risk from PAHs (**a**), OXY- (**b**) and NITRO-PAHs (**c**) in ultrafine, fine and coarse fraction of PM in the four sampling sites.

Figure 9 shows percentage distribution of CR from PAHs and derivatives associated with ultrafine, fine and coarse fractions of PM. In RM and WWTP sites, despite the greater abundance of organic compounds in the ultrafine fraction (Section 3.2—Figure 4), the higher CR percentage was obtained in the fine fraction. This is probably due to the compound distribution in the three PM fractions, since CR estimation depends on a single compound parameter as shown in Section 2.3. In the FEED and COW sites, CR shows a homogeneous size distribution with slightly higher values in coarse fractions in agreement with CR values comparable to each other and therefore equally distributed in the three PM sizes.

**Figure 10:**
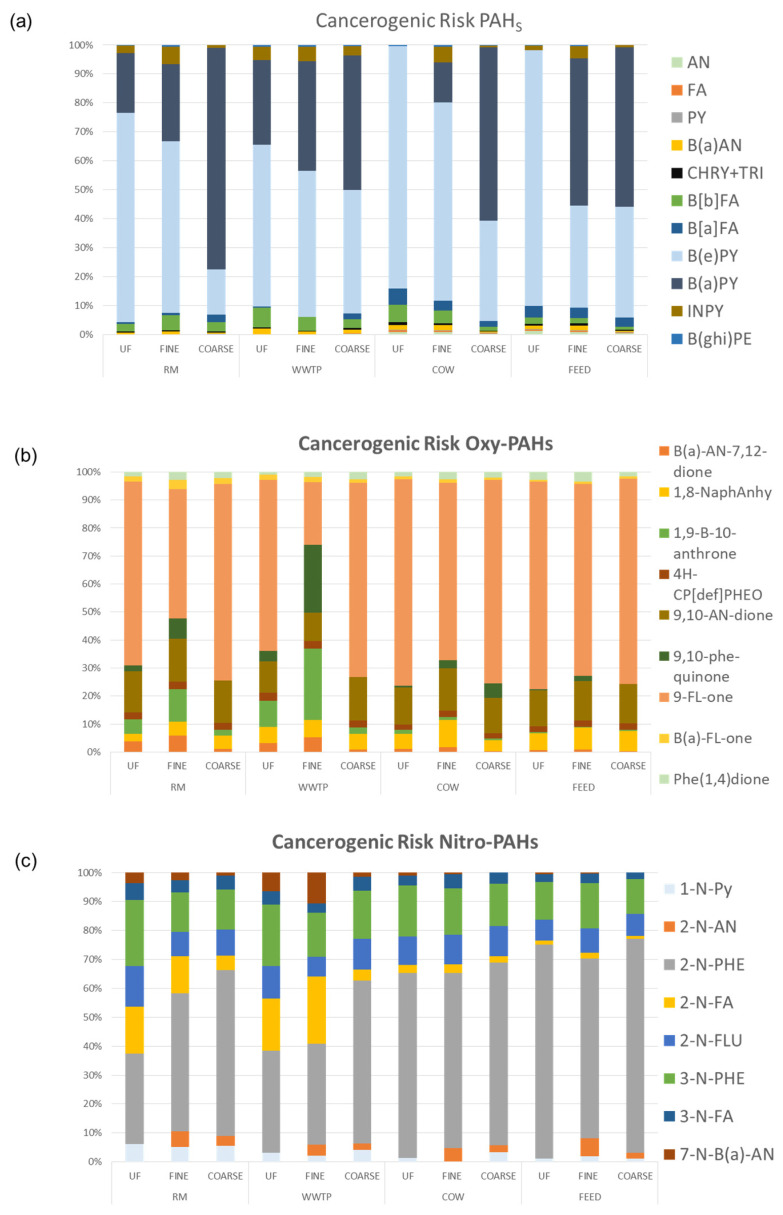
Percentage distribution of carcinogenic risk of PAHs (**a**), Oxy- (**b**) and Nitro-PAHs (**c**) in ultrafine, fine and coarse fraction of PM in the four sampling sites.

Figure 10 shows CR percentage associated with each single compound in ultrafine, fine and coarse fractions of PM for the study sites. As regards PAHs, 90% of CR is related to HMWPAHs in all three PM fractions and for the all sites. B(a)PY and B(e)PY are the compounds that most contribute to risk levels. As known, wood and fuel combustion accounts for over 75% on benzopyrene concentration measured in PM [35,36,37], therefore the correlation highlighted between CR values and these compounds, confirms the presence of pyrolytic sources, mainly vehicle exhaust and biomass burning, such as combustion of grassland, brushwood and agricultural wastes, vegetation and forest fires, as also shown in PCA. Furthermore, in urban areas the heating systems are switched off during the spring period, so the combustion of biomass and fuel becomes the predominant pyrolytic source [38]. Nevertheless, for all the sites CR values associated with this class of compounds are lower than those reported by other authors [39]. 

Regarding Oxy-PAHs, higher CR percentages are associated with 9-FL-one, 9,10-AN-dione and B(a)-FL-one in all the areas and for all the PM fractions. In addition, in RM and WWTP sites, 1,9-B-10-Anthrone and 9-10-phe-quinone contribute significantly to the risk assessment in fine and ultrafine fractions. This further proves that the human health risk from combustion sources is not negligible in any study areas.

As for Nitro-PAHs, 2-N-Phe, 3-N-Phe and 2-N-Flu are PAH derivatives most responsible for CR in all sites. In RM and WWTP sites, in fine and ultrafine fractions, risk induced by 2-N-Fa was significant, as well. As these compounds can be produced in combustion processes or from photochemical reactions of native PAHs in atmosphere [40,41,42,43], their greatest contribution to the risk can have both primary origin for diesel exhaust particles and secondary origin for atmospheric transformations of contaminants.

### 4.2. PCB 

**Figure 11:**
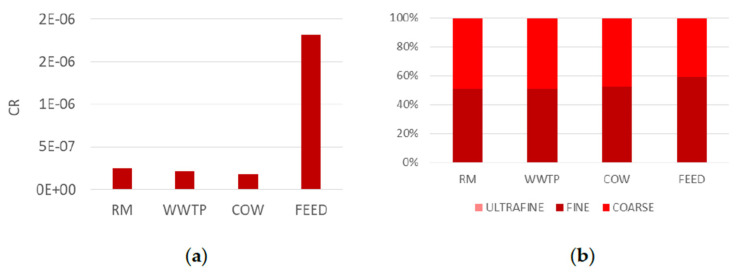
Carcinogenic risk due to PM10 bound dioxin-like PCB (panel **a**) and their distribution in the three size fractions of PM (panel **b**).

Figure 11a shows CR due to PM10 bound dioxin-like PCB in the study sites. CR ranges from 1.8 × 10^−7^ to 1.8 × 10^−6^. The highest CR value is obtained in the FEED site probably due to PCB contamination often detected in feed and seeds as also shown by other authors [29]. Commonly, PCB emissions may be affected by the industries and chemical plants [44], but, as the site is located in a rural area, near the coast and away from industrial areas, this finding can be due to initial feed contamination. Furthermore, in this site, CR is lightly greater than the USEPA [12] acceptable level (1 × 10^−6^) chosen as a reference value and by no means negligible since it was determined considering only the inhalation pathway. Moreover, it is greater than the carcinogenic risk calculated in municipal solid waste landfill where PCBs are emitted from outdated and damaged equipment [45]. For the outdoor urban site, the CR value related to PCBs is below the USEPA limit threshold and comparable with other studies [44].

In addition, CR percentage distribution in the three size fractions of PM (Figure 11b) highlights a human health risk mainly due to fine and coarse fractions. CR contribution of ultrafine fraction is about 0.16% in all the sites. Since the only dioxin-like PCBs detected in the ultrafine fractions are PCB105 and 187 and their concentration was about 10^−6^ µg/m^3^, their contribution to CR appeared to be negligible.

**Figure 12:**
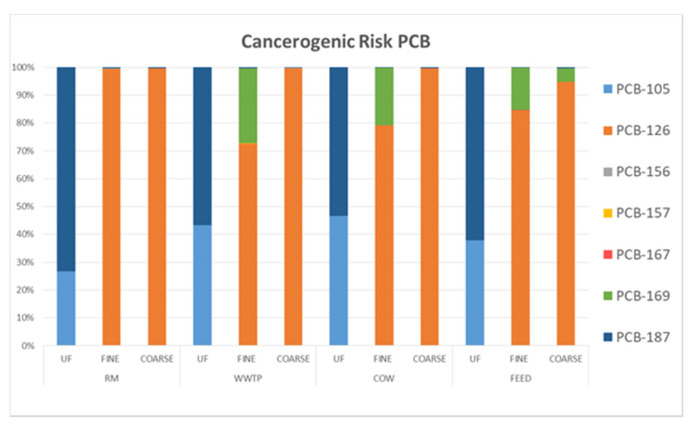
Percentage distribution of carcinogenic risk of each single compound in ultrafine, fine and coarse fraction of PM in the four sampling sites.

Figure 12 shows CR percentage due to each dioxin-like PCBs. As already pointed out, PCB 105 and 187 affected the values of CR in UF fractions. In F and C fractions, CR levels were mainly related to PCB 126 and 169. PCB 126 can be considered the most potent dioxin-like PCB and most abundant congener in ambient air [46]. As known, the main sources of PCBs are combustion processes, for example waste incineration, industrial transformation activities, fossil fuel burning and road transport [47,48,49], therefore the correlation between CR and dioxin-like PCBs confirms combustion as predominant source of pollution for WWTP and RM in agreement with their location.

In addition, Lohmann et al. [50] highlighted that non-ortho substituted PCBs, as PCB 126 and 169, are absorbed more strongly on the carbon particles than ortho substituted PCBs and more than 50% of these compounds is detected in marine aerosol samples. Marine aerosol could influence the study areas, since COW and FEED sites are located close to Mediterranean coast and RM and WWTP sites are subject to winds from the sea.

### 4.3. Total Carcinogenic Risk

**Figure 13:**
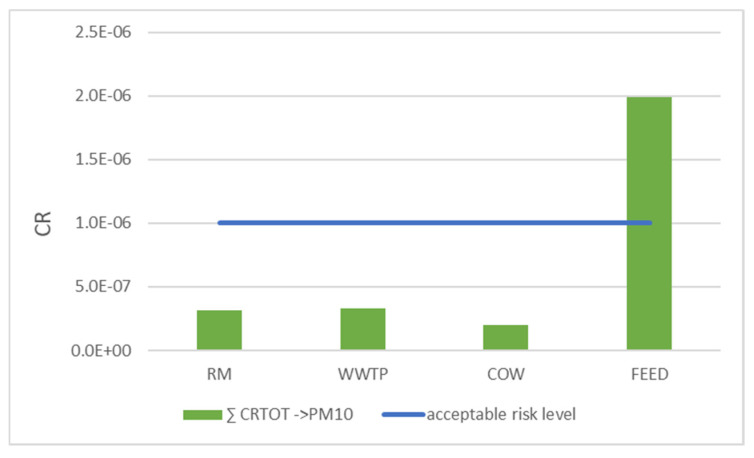
Total carcinogenic risk obtained as sum of CR associated with PAHs, Nitro-PAHs, Oxy-PAHs and dioxin-like PCBs in PM10 and for all sites.

Figure 13 shows the total carcinogenic risk obtained as sum of CR associated with 11 PAHs, 9 Nitro-PAHs, 9 Oxy-PAHs and 7 dioxin-like PCBs in PM10 and for all sites. Total CR was compared to 1 × 10^−6^, value suggested by USEPA as an acceptable risk level (Figure 13—blue line).

For RM, WWTP and COW sites, CR values were lower than those set by USEPA, highlighting a low level of risk in these areas. However, these values could be underestimated as the contribution of some compounds, whose related parameters were neither available nor obtainable from chemically similar substances, was neglected. 

In the FEED site, the total CR value is between the acceptable risk level (1 × 10^−6^) and the tolerable one (1 × 10^−4^) [23,24]. However, as with the other sites, this value may also be underestimated. In this site, a high amount of airborne dust was detected since feed grinding is continuously carried out in situ. Moreover, the feed is frequently moved to supply the numerous feeders in the stables of livestock plant. The presence of high quantities of volatilized feed containing toxic substances (such as PCB, PBDE and NBFR) and the use of tractors, direct sources of PAH and derivatives, can justify higher CR values. Therefore, workers involved in the handling of feed and seeds, could be subject to a no negligible carcinogenic risk despite the tolerable value is not exceeded. In addition, CR was calculated by determining only the contribution related to the inhalation route, but ingestion and dermal contact of dusts can be significant exposure pathways. Therefore, further studies have to be developed to evaluate the carcinogenic risk due to the three exposure pathways.

### 4.4. PBDE and NBFR 

**Figure 14:**
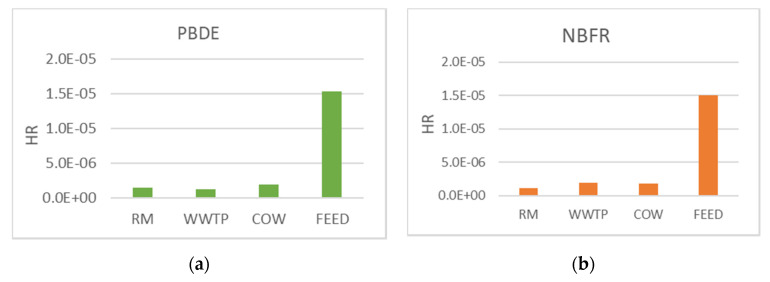
Non-carcinogenic risk due to PM10 bound PBDE (**a**) and NBFR (**b**) in all the monitoring sites.

Figure 14 shows HR due to PM10 bound PBDE and NBFR in the study sites. 

As described in Section 2.3, HR was calculated using the reference concentrations (RfC) associated with each analyte. For NBFR these data are lacking and it was decided to use the highest RfC value associated with PBDE as a precautionary measure for public health safety. This was possible due to the chemical–physical similarity between the two classes of compounds, as reported by other authors [25].

HR ranges from 1 × 10^−6^ to 1.5 × 10^−5^ and all values were well below the safe level equal to 1 (Section 2.3), suggesting that non-carcinogenic risk could not occur. Nevertheless, significant differences could be observed between sites. 

As already shown by PCB for CR, the highest HR value was obtained in the FEED site for both PBDE and NBFR probably due to contamination often detected in feed and seeds at non-negligible levels as a result of manufacturing processes [34,51]. In addition, for PBDE, HR values were lower than the ones reported by other authors for outdoor environments. Conversely, for NBFR few works of risk assessment are focused on outdoor sites and many studies are aimed at evaluating HR associated with inhalation exposure of settled dust in indoor environments such as homes and offices [52].

**Figure 15:**
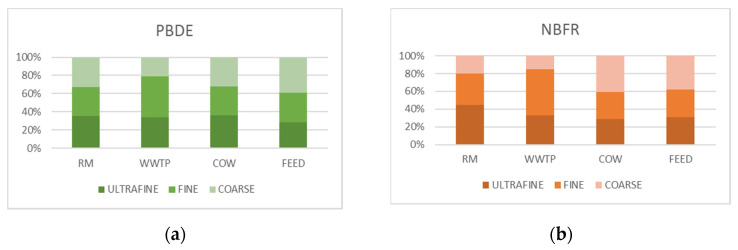
Percentage distribution of non-carcinogenic risk from PBDE (**a**) and NBFR (**b**) associated with ultrafine, fine and coarse fractions of PM for all the sites.

**Figure 16:**
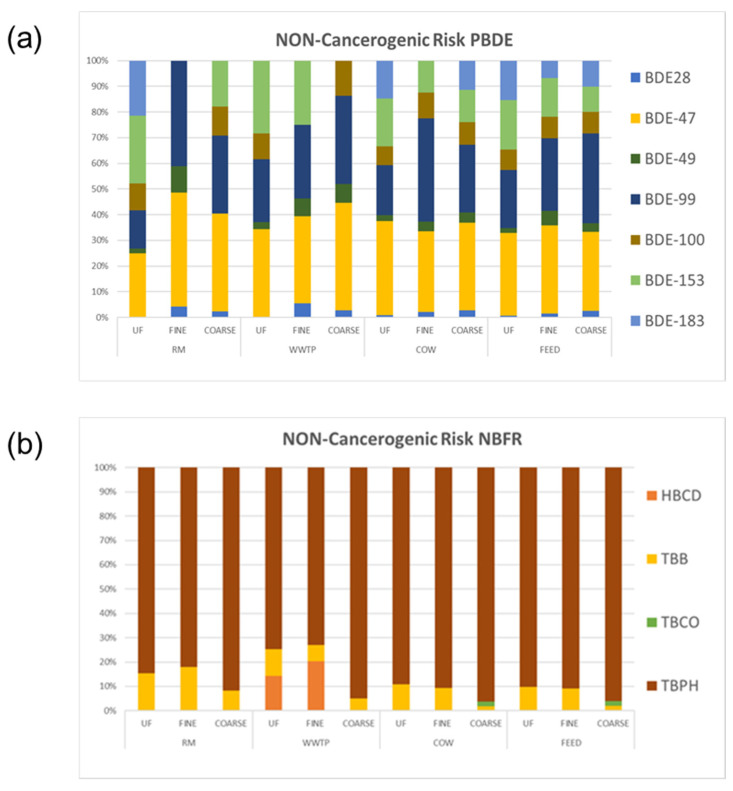
Non-carcinogenic risk percentage associated with each single compound in ultrafine, fine and coarse fractions of PM for all the sites. (**a**). Non-Cancerogenic Risk PBDE. (**b**) Non-Cancerogenic Risk NBFR.

Figure 15 shows percentage distribution of HR from PBDE and NBFR associated with ultrafine, fine and coarse fractions of PM in the study areas. As for PBDE, in WWTP about 85% of HR was related to fine and ultrafine fractions, whereas a homogeneous distribution between the fractions occurs in the other sites. As regards NBFR, HR was predominantly associated with fine and ultrafine fractions of PM in both WWTP and RM and evenly distributed in COW and FEED. As highlighted in Section 3.3.2, no specific source of PBDE and NBFR was identified at the WWTP and RM sites. However, bubble bursting at the air–water interface in the aeration tanks of WWTP may strongly contribute to the formation of aerosols containing flame retardants [53,54]. In addition, some NBFR can accumulate in dewatered sludge during wastewater treatment processes [55]. Lastly, the replacement of PBDE mixtures with NBFR can be considered a probable source of these compounds in urban air [56]. The different percentage profile of HR obtained in the study sites for the three PM fractions can be due to a different distribution of compounds. Figure 16 shows HR percentage associated with each single compound in ultrafine, fine and coarse fractions of PM for the study areas.

In all the sites and for all size fractions of PM, HR levels were related to PBDE according the following order: BDE-47 > BDE-99 > BDE-153 > BDE-100 >BDE-183. These compounds were present in the commercial formulations penta- and octa-BDE: BDE47 and 99 were the most abundant in the technical penta-BDE and BDE183 was the dominating congener of the technical octa-BDE [57]. About 90% of HR was due to the congeners of technical penta-BDE suggesting that, despite the phasing out of PBDEs, outdated products still in use may be a source of non-carcinogenic risk to public health. Likewise, HR due to BDE183 was related to the use of products containing the octa-BDE mixture. In addition, combustion processes can also be considered a source of PBDE as reported by some authors that detected these chemicals in environmental samples such as road dust, green residues, etc. [58,59].

As concerns NBFR, at all the sites and for all the PM fractions HR was mostly associated with TBPH accounting for about 80–90% of total HR. Furthermore, TBB shows a significant correlation with HR, as well. Both these compounds were the most abundant flame retardants in the commercial formulation named Firemaster 550, which replaced the technical penta-BDE mixture [56]. Lastly, a non-negligible contribution of HBCD to HR levels can be observed for fine and ultrafine fractions in WWTP. As previously reported, HBCD is among NBFRs that can be adsorbed on suspended particulate matter and accumulated in sewage sludge because of its high hydrophobicity [55]. 

### 4.5. Total Non-Carcinogenic Risk

**Figure 17:**
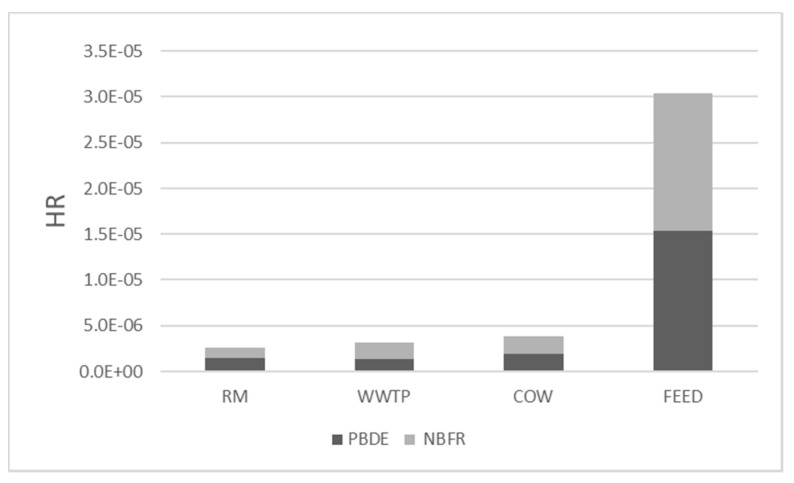
Total non-carcinogenic risk obtained as sum of HR associated with PBDE and NBFR in PM10 and for all sites.

Figure 17 shows the total non-carcinogenic risk obtained as sum of HR associated with PBDE and NBFR in PM10 and for all sites. The comparison between total HR and the safe level equal to 1, suggested by USEPA [12] as an acceptable level for protection of public health, shows that no risk can occur for the people operating in these sites. Nevertheless, FEED site requires attention since HR value is an order of magnitude higher than the ones found for the other sites. Since the HR values could also be underestimated, as already underlined for the carcinogenic risk, further safeguards for workers should be considered during feed handling activities. Furthermore, beyond the inhalation route, ingestion and dermal contact of dust can be significant exposure pathways; therefore, further studies have to be carried out to evaluate public health risk level due to these exposure routes.

Lastly, PBDEs contributed largely to the total non-carcinogenic risk in all the study sites. This finding suggests that outdated products are currently widely spread. Therefore, PBDEs are still a source of risk to public health, although their use has been banned in the European Union since 2008.

## 5. Conclusions

Since epidemiological studies mainly link the carcinogenic effects of exposure to UF, F and coarse PM emissions to the organic fraction, organic speciation for the three fractions of aerosols in three workplaces (WWTP, FEED and COW) and in an urban one (RM) was carried out. Belonging to six different classes of persistent organic pollutants 66 organic compounds were determined in the PM size fractions. To identify the possible characteristic emissive sources of contaminants, the principal component analysis was applied. PCA highlighted specific correlations between WWTP and RM showing that the main WWTP source was urban source. FEED and COW were characterized by specific emission sources showing, in the FEED site, high concentrations of organic contaminants. In addition, both carcinogenic and non-carcinogenic risks were estimated to assess the extent of threat to which workers were exposed. Even if at all the sites and for all the size fractions of PM both carcinogenic and non-carcinogenic risk values were well below tolerable and safe levels of risks recommended by the regulatory agencies, the FEED site highlighted a total CR value between the acceptable risk level (1 × 10^−6^) and the tolerable one (1 × 10^-4^). Therefore, particular attention should be paid by workers involved in the handling of feed and seeds. Lastly, the detection of NBFR at not negligible concentrations, address research towards a deeper study of the toxicological behavior of these compounds to identify specific parameters to be used for risk value determination and suitable to a sound risk assessment.

## Figures and Tables

**Table 1:** Description of the study sites.

Monitoring Site	Type of Site	Principal Emission Sources
RM	Urban	Traffic, residential housing, human activities.
WWTP	Industrial/Suburban	Traffic, residential housing, wastewater treatment plant activities.
COW	Industrial/Rural	Livestock shelter and feeders, agricultural tractors.
FEED	Industrial/Rural	Grinding, mixing, and storing of feeds, feed handling, agricultural tractors

RM: Urban site. WWTP: Wastewater treatment plant. COW: Shed site. FEED: Feed site

## Supplementary Materials

The following are available online at https://www.mdpi.com/article/10.3390/ijerph18084352/s1, Table S1: Organic compound list and codes used in PCA development, Table S2: PAH parameters for carcinogenic risk assessment, Table S3: Oxy-PAH parameters for carcinogenic risk assessment, Table S4: Nitro-PAH parameters for carcinogenic risk assessment, Table S5: PCB parameters for carcinogenic risk assessment, Table S6: PBDE parameters for non-carcinogenic risk assessment, Table S7: title, Table S1: NBFR parameters non carcinogenic risk assessment.
