# Supplementary material for: Toxic Organic Contaminants in Airborne Particles: Levels, Potential Sources and Risk Assessment
Source: Int J Environ Res Public Health. 2021 Apr 20;18(8):4352. doi: 10.3390/ijerph18084352 (PMC8073354; doi:10.3390/ijerph18084352)
Supplement: Supplementary file 1 [file ijerph-18-04352-s001.zip › ijerph-1168532-supplementary.pdf]

Table S1. Organic compound list and codes used in PCA development

	Compond	*PCA code*	*Abbreviation*
PAHs	Anthracene	P1	
	Fluoranthene	P2	
	Pyrene	P3	
	Benz[a]anthracene	P4	
	Chrysene +Trif	P5	
	Benz[k]fluoranthene	P6	
	Benz[b]fluoranthene	P7	
	Benz[a]fluoranthene	P8	
	Benz[e]pyrene	P9	
	Benz[a]pyrene	P10	
	Benz[ghi]perylene	P11	
	Indeno[1,2,3-cd]pyrene	P12	
	Coronene	P13	
Oxy-PAHs	9-Fluorenone	O1	
	1,8-Naphthalic anhydride	O2	
	4H-Cyclopenta[def]phenanthrene-4-one	O3	
	1,4-Phenanthrenedione	O4	
	9,10-Anthracene-dione	O5	
	9,10-Phenanthrenequinone	O6	
	Benz[a]fluorenone	O7	
	1,9-Benz-10-anthrone	O8	
	Benzo[a]anthracene-7,12-dione	O9	
Nitro-PAHs	2-N-Fluorene	N1	
	2-N-Anthracene	N2	
	2-N-Phenanthrene	N3	
	3-N- Phenanthrene	N4	
	9-N- Anthracene	N5	
	9-N- Phenanthrene	N6	
	2-N-Fluoranthene	N7	
	1-N-Pyrene	N8	
	3-N-Fluoranthene	N9	
	7-N-benz[a]anthracene	N10	
PCBs	2,4,4′-Trichlorobiphenyl	PB28	PCB28
	2,3,3′,4,4′-Pentachlorobiphenyl	PB105	PCB105
	3,3',4',4',5 Pentachlorobiphenyl	PB126	PCB126
	2,2′,3,4,4′,5′-Hexachlorobiphenyl	PB138	PCB138
	2,2',3,4',5,5'-Hexachlorobiphenyl	PB146	PCB146
	2,2′,3,5,5′,6-Hexachlorobiphenyl	PB151	PCB151
	2,3,3′,4,4′,5-Hexachlorobiphenyl	PB156	PCB156
	2,3,3',4,4',5'-Hexachlorobiphenyl	PB157	PCB157
	2,3',4,4',5,5'-Hexachlorobiphenyl	PB167	PCB167
	3,3',4,4',5,5'-Hexachlorobiphenyl	PB169	PCB169
	2,2′,3,3′,4,4′,5-Heptachlorobiphenyl	PB170	PCB170
	2,2',3,3',4',5,6-Heptachlorobiphenyl	PB177	PCB177
	2,2',3,4,4',5',6-Heptachlorobiphenyl	PB183	PCB183
	2,2',3,4',5,5',6-Heptachlorobiphenyl	PB187	PCB187
	2,3,3',4,4',5,6-Heptachlorobiphenyl	PB190	PCB190
PBDEs	2,4,4'-Tribromodiphenyl ether	B28	BDE28
	2,2',4,4'-Tetrabromodiphenyl ether	B47	BDE47
	2,2',4,5'-Tetrabromodiphenyl ether	B49	BDE49
	2,2′,4,4′,5-Pentabromodiphenyl ether	B99	BDE99
	2,2′,4,4′,6-Pentabromodiphenyl ether	B100	BDE100
	2,2',4,4',5,5'-Hexabromodiphenyl ether	B153	BDE153
	2,2',3,4,4',5',6-Heptabromodiphenyl ether	B183	BDE183
NBFRs	2,4-Dibromophenol	NB1	2,4-DBP
	2,6-Dibromophenol	NB2	2,6-DBP
	2,4,6-Tribromophenol	NB3	TBP
	Allyl-2,4,6-tribromophenyl ether	NB4	ATE
	1,2-dibromo-4-(1,2-dibromoethyl) cyclohexane	NB5	TBECH

2,3,4,5,6-pentabromoethylbenzene	NB6	PBEB
2,3-dibromopropyl-2, 4, 6-tribromophenylether	NB7	DPTE
Hexabromobenzene	NB8	HBB
2-Ethylhexyl-2,3,4,5-tetrabrombenzoate	NB9	TBB
1,2-bis(2,4,6-tribromophenoxy) ethane	NB10	BTBPE
bis (2-ethylhexyl)-3,4,5,6-tetrabromo-phthalate	NB11	TBPH

Table S2. PAH parameters for carcinogenic risk assessment

	Compond	*DET (h)*[a]	*EF (d)*	*ED(y)*	*AT (y)*	*IUR (µg/m³)*[1,2]
PAHs	Anthracene	8	350	30	70	1,10E-05
	Fluoranthene	8	350	30	70	1,10E-06
	Pyrene	8	350	30	70	1,10E-06
	Benz[a]anthracene	8	350	30	70	1,10E-04
	Chrysene +Trif	8	350	30	70	1,10E-05
	Benz[b]fluoranthene	8	350	30	70	1,10E-04
	Benz[a]fluoranthene	8	350	30	70	1,10E-04[b]
	Benz[e]pyrene	8	350	30	70	1,10E-03[c]
	Benz[a]pyrene	8	350	30	70	1,10E-03
	Indeno[1,2,3-cd]pyrene	8	350	30	70	1,10E-04
	Benz[ghi]perylene	8	350	30	70	1,10E-05

[a] Daily working hours; [b] Benz[b]fluoranthene value (see par. 2.3); [c] Benz[a]pyrene value (see par. 2.3)

Table S3. Oxy-PAH parameters for carcinogenic risk assessment

	Compond	*DET (h)*	*EF (d)*	*ED(y)*	*AT (y)*	*IUR (µg/m³)*[3]
Oxy-PAHs	9-Fluorenone	8	350	30	70	8,70E-05
	1,8-Naphthalic anhydride	8	350	30	70	8,70E-05
	4H-Cyclopenta[def]phenanthrene-4-one	8	350	30	70	8,70E-05
	1,4-Phenanthrenedione	8	350	30	70	8,70E-05
	9,10-Anthracene-dione	8	350	30	70	8,70E-05
	9,10-Phenanthrenequinone	8	350	30	70	8,70E-05
	Benz[a]fluorenone	8	350	30	70	8,70E-05
	1,9-Benz-10-anthrone	8	350	30	70	8,70E-05
	Benzo[a]anthracene-7,12-dione	8	350	30	70	8,70E-05

Table S4. Nitro-PAH parameters for carcinogenic risk assessment

	Compond	*DET (h)*	*EF (d)*	*ED(y)*	*AT (y)*	*IUR (µg/m³)*[3]
Nitro-PAHs	2-N-Fluorene	8	350	30	70	8,70E-05
	2-N-Anthracene	8	350	30	70	8,70E-05
	2-N-Phenanthrene	8	350	30	70	8,70E-05
	3-N- Phenanthrene	8	350	30	70	8,70E-05
	9-N- Anthracene	8	350	30	70	8,70E-05
	9-N- Phenanthrene	8	350	30	70	8,70E-05
	2-N-Fluoranthene	8	350	30	70	8,70E-05
	1-N-Pyrene	8	350	30	70	8,70E-05
	3-N-Fluoranthene	8	350	30	70	8,70E-05
	7-N-benz[a]anthracene	8	350	30	70	8,70E-05

Table S5. PCB parameters for carcinogenic risk assessment

	Compound	DET (h)	EF (d)	ED(y)	AT (y)	IUR (µg/m³)[4]
PCBs	PB105	8	350	30	70	1,10E-03
	PB126	8	350	30	70	3,80E+00
	PB156	8	350	30	70	1,10E-03
	PB157	8	350	30	70	1,10E-03
	PB167	8	350	30	70	1,10E-03
	PB169	8	350	30	70	1,10E+00
	PB187	8	350	30	70	1,10E-03

Table S6. PBDE parameters for non-carcinogenic risk assessment

	Compound	DET (h)	EF (d)	ED(y)	AT (y)	RFC (µg/m³)[6]
PBDEs	BDE28	8	350	30	25	1,10E-02[d]
	BDE47	8	350	30	25	1,10E-02[d]
	BDE49	8	350	30	25	1,10E-02[d]
	BDE99[5]	8	350	30	25	7,00E-03
	BDE100[5]	8	350	30	25	7,00E-03
	BDE153	8	350	30	25	7,00E-03
	BDE183	8	350	30	25	1,10E-02[d]

[d] as a precautionary approach, the highest value associated with PBDEs was chosen (see par. 4.4)

Table S7. NBFR parameters non carcinogenic risk assessment

	Compound	DET (h)	EF (d)	ED(y)	AT (y)	RFC (µg/m³)[e]
NBFRs	HBCD	8	350	30	25	1,10E-02
	TBB	8	350	30	25	1,10E-02
	TBCO	8	350	30	25	1,10E-02
	TBPH	8	350	30	25	1,10E-02

[e] as a precautionary approach, the highest value associated with PBDEs was chosen (see par. 4.4)

1. EPA (2003) Integrated risk information system. http://www. epa.gov/iris. Accessed 16 June 2010
2. OEHHA, 2002, 2005. Air Toxics Hot Spots Program Risk Assessment Guidelines. Part II: Technical Support Document for Describing Available Cancer Potency Factors. Office of Environmental Health Hazard Assessment. Available from: /http://www.oehha.ca.gov/air/hot_spots/pdf/TSDNov2002. pdfS (p. 109) and /http://www.oehha.ca.gov/air/hot_spots/ pdf/May2005Hotspots.pdfS (p. 8 and A-1).
3. WHO. Air quality guidelines for Europe. Copenhagen, Denmark:WHO Regional Office for Europe; 2000 http://www.euro.who.int/__data/assets/pdf_file/0005/74732/E71922.pdf acessed March 19, 2013].
4. OEHHA. 2011. Office of Environmental Health Hazard Assessment (OEHHA). Appendix A: Hot Spots Unit Risk and Cancer Potency Values Updated October 2020. https://oehha.ca.gov/media/downloads/crnr/appendixa.pdf
5. EPA Region 9, 2019. Toxicity and chemical/physical properties for Regional Screening level (RSL) of Chemical Contaminants at Superfund Sites. [Online] http://www.epa.gov/region9/superfund/prg/. US Environmental Protection Agency.
6. INAIL (2020) PBDE Monograph. [Online] https://www.inail.it/cs/internet/docs/alg-pubbl-polibromodifenileteri-pbde-microinqunanti-organici.pdf. Italian Workers 'Compensation Authority.
